# A comprehensive treatment analysis of primary squamous cell carcinoma of the prostate with inguinal lymph node metastasis: a case report and literature review

**DOI:** 10.3389/fonc.2026.1761392

**Published:** 2026-03-03

**Authors:** Hongwei Li, Xiaoqing Yang, Xibin Liu, Zhuoqun Wang, Lei Xu, Zi Che

**Affiliations:** 1 Department of Pathology, Zibo Central Hospital, Shandong, Zibo, China; 2 Department of Urology, Zibo Central Hospital, Shandong, Zibo, China; 3 Department of Medical Oncology, Zibo Central Hospital, Shandong, Zibo, China

**Keywords:** chemo-radiotherapy, inguinal lymph node metastasis, long-survival, primary, prostate cancer, squamous cell carcinoma

## Abstract

**Introduction:**

Primary squamous cell carcinoma(SCC) of the prostate is a rare clinical condition, characterized by diagnostic challenges in the early stages, a high potential for metastasis, considerable malignancy, and a poor prognosis. Reporting such cases may contribute to a better understanding of the pathogenesis and clinical manifestations of prostatic SCC, as well as facilitate the exploration of diagnostic approaches and effective treatment strategies.

**Case presentations:**

A retrospective analysis was conducted on the clinical case of a 62-year-old male patient with primary prostatic SCC and concurrent inguinal lymph node metastasis, admitted to our institution. The diagnosis was confirmed by histopathological examination following fine-needle aspiration biopsy. The patient underwent two cycles of intravenous chemotherapy, followed by radical surgical resection and adjuvant radiotherapy. By integrating findings from this case with current literature, this study aims to elucidate the etiology, clinical manifestations, diagnostic strategies, therapeutic modalities, and prognostic factors associated with this rare malignancy.

**Conclusion:**

The patient demonstrated good tolerance to both chemotherapy and radiotherapy, with a significant reduction in lesion size observed following treatment. After comprehensive management including surgical resection, the patient has remained alive for nearly two years of continuous follow-up. Notably, prostatic SCC exhibits poor responsiveness to endocrine therapy. The most effective therapeutic approach involves early radical surgery, which, when integrated with chemotherapy, radiotherapy, and other modalities, may contribute to prolonged survival.

## 1 Introduction

Prostate cancer is the second most commonly diagnosed malignant neoplasm in men worldwide. According to the 2020 estimates from GLOBOCAN, there were 1,514,259 new cases and 375,304 prostate cancer–related deaths globally, with a marked increasing trend in incidence observed in China (1, 2). Primary squamous cell carcinoma of the prostate is a rare pathological subtype of prostate cancer, accounting for approximately 0.5% to 1% of all prostate cancer cases (3). Unlike other forms of prostate cancer, prostatic SCC is characterized by high malignancy, aggressive invasiveness, a strong propensity for metastasis, and an overall poorer prognosis (3, 4). While bone metastasis is commonly observed in prostate cancer, inguinal lymph node metastasis is relatively uncommon. The paucity of reported prostatic SCC cases in both Chinese and international literature has led to a lack of standardized clinical treatment protocols. In February 2022, a patient with a prostate tumor was admitted to our institution. The postoperative pathological examination confirmed a diagnosis of prostatic SCC. This report presents the clinical features, diagnostic evaluation, treatment course, and a comprehensive review of the relevant literature.

## 2 Case presentations

A 62-year-old male presented to our outpatient clinic with progressive difficulty in urination. Laboratory evaluation revealed a total prostate-specific antigen (tPSA) level of 4.72 ng/mL. Physical examination demonstrated a hard right lobe of the prostate and a palpable enlarged lymph node measuring approximately 2.0 × 1.8 × 1.6 cm in the left inguinal region. Notably, the patient had undergone a subtotal gastrectomy four years prior for gastric malignancy, with histopathological findings confirming a middle-to-low differentiated adenocarcinoma of the antrum, ulcerative type. He had been under regular follow-up postoperatively, remained asymptomatic, and showed no evidence of recurrence. Given these clinical findings, the possibility of prostatic neoplasm could not be excluded; therefore, the patient was admitted for further diagnostic evaluation and management.

**Figure 1:**
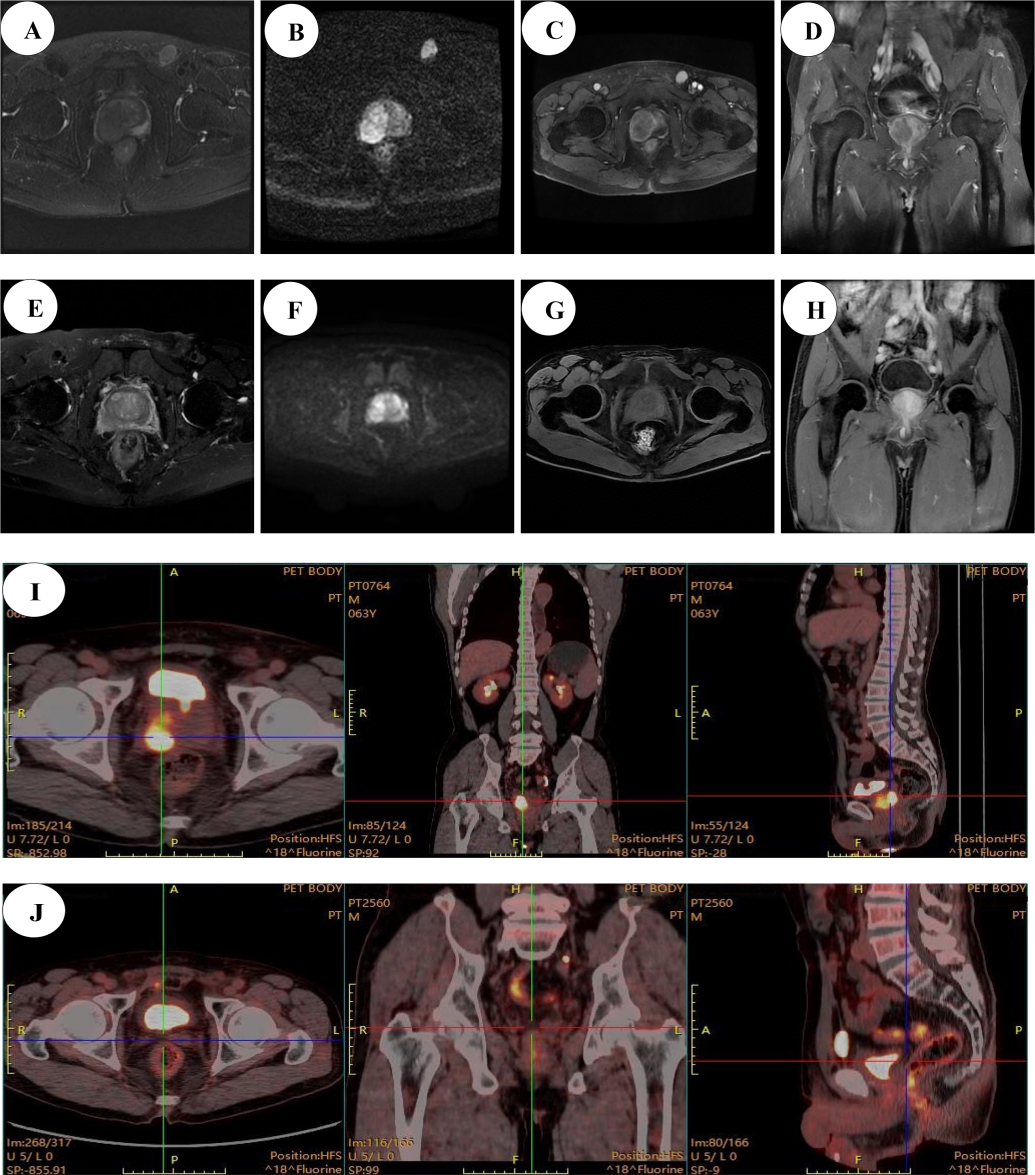
Imaging manifestations of the patient before and after treatment MRI Images: **(A–D)** pre-chemotherapy images; **(E–H)** post-chemotherapy images (prostate volume reduced by approximately 33.7%); **(A)** AX T2 FS FSE; **(B)** AX DWI; **(C)** AX LAVA-XV+C; **(D)** Cor LAVA-XV; **(E)** AX T2 FS FSE; **(F)** AX DWI; **(G)** AX LAVA-XV+C; **(H)** Cor LAVA-XV PET⁃CT images: **(I)** chemotherapy and pre-surgery PET-CT images; **(J)** chemotherapy and post-radical surgery PET-CT images.

The patient underwent a comprehensive panel of laboratory tests, including tumor marker assessments, with the following results: carcinoembryonic antigen (CEA) 6.93 ng/mL, carbohydrate antigen (CA)-125 14.10 U/mL, CA-724 2.92 U/mL, CA19-9 166.00 U/mL, tPSA 3.69 ng/mL, free PSA (fPSA) 0.33 ng/mL (fPSA/tPSA ratio: 8.9%), neuron-specific enolase (NSE) 9.15 ng/mL, and alpha-fetoprotein (AFP) 2.32 ng/mL. A multiparametric prostate magnetic resonance imaging (MRI) examination was performed. The prostate measured approximately 5.7×4.1×4.9 cm. MRI revealed heterogeneous signal intensity within the gland, with multiple nodular foci of variable size in the central and transition zones, isointense on T1WI and mildly hyperintense on fat-suppressed T2WI. Notably, a 3.8 × 2.8 cm nodular lesion was identified at the 6- to 11-o’clock position in the right transition zone, demonstrating an incomplete capsule, ill-defined margins, isointensity on T1WI, hypointensity on T2WI, and mild hypointensity on DWI. During the early dynamic contrast-enhanced phase, the lesion exhibited marked but heterogeneous enhancement, with evidence of invasion into the right peripheral zone, resulting in a PI-RADS version 2.1 score of 5. Bilateral seminal vesicles were distended with heterogeneous signal intensity, while the bladder was poorly distended and showed mild wall thickening. The rectum appeared unremarkable, and small pelvic lymph nodes were present without ascites or suspicious features. Additionally, a left inguinal nodule measuring approximately 1.4 × 1.9 cm demonstrated isointense T1 and mildly prolonged T2 signal, with pronounced enhancement on post-contrast imaging (**Figure 1A–D**).

Following the initial diagnosis, the patient underwent an ultrasound-guided transperineal prostate biopsy and excision of the left inguinal lymph node. Histopathological examination revealed that the prostatic tissue consisted of moderately to poorly differentiated squamous cell carcinoma with focal necrosis. Similarly, the left inguinal lymph node showed metastatic involvement by moderately to poorly differentiated squamous cell carcinoma with focal necrosis. Given the rarity of prostatic squamous cell carcinoma presenting with inguinal lymph node metastasis, a comprehensive physical evaluation was performed to exclude secondary primary malignancies. This included cystoscopy and clinical assessment for potential squamous cell carcinomas in the head and neck region, skin, oral cavity, bladder, and penis; no suspicious lesions were identified. Subsequently, the patient underwent 18F-fluorodeoxyglucose positron emission tomography/computed tomography (18F-FDG PET-CT) for systemic staging. The imaging demonstrated the following findings: (1) Heterogeneous density within the prostate with markedly increased FDG metabolism (SUVmax: 9.89), consistent with a malignant prostatic lesion; additionally, a right inguinal lymph node (1.2 × 0.6 cm) exhibited elevated FDG uptake (SUVmax: 5.53), suggestive of metastatic involvement; (2) Post-gastrectomy changes without metabolic evidence of recurrence at the anastomotic site (**Figure 1I**).

Primary prostatic SCC is an exceedingly rare clinical entity, with limited case reports documented in both domestic and international literature and a lack of standardized treatment guidelines. Primary prostatic SCC is intrinsically resistant to androgen deprivation therapy. Given the absence of evidence-based, disease-specific chemotherapy guidelines for prostatic SCC, treatment is empirically guided by established regimens for extraprostatic squamous cell carcinomas—particularly those of the lung, head and neck, and esophagus—where platinum-based combination chemotherapy remains the standard of care. Commonly adopted regimens include paclitaxel or docetaxel plus cisplatin/carboplatin (TP/CP), etoposide plus cisplatin (EP), gemcitabine plus cisplatin/carboplatin (GP), and 5-fluorouracil plus cisplatin. In this case, neoadjuvant chemotherapy was administered with curative intent to achieve tumor downstaging and improve surgical resectability. Following multidisciplinary oncology consultation and informed consent from the patient and family, two cycles of intravenous chemotherapy were delivered: cisplatin (70 mg/m²) administered as a divided infusion on days 1–3, and nanoparticle albumin-bound paclitaxel (240 mg/m²) administered on days 1 and 8. No grade ≥2 toxicities were observed per the Common Terminology Criteria for Adverse Events (CTCAE) v5.0; specifically, hematologic (neutropenia, anemia, thrombocytopenia), gastrointestinal (diarrhea, nausea/vomiting), dermatologic (rash), and neurologic (peripheral sensory neuropathy) adverse events were all limited to grade 1. The regimen was well tolerated, with no treatment delays or dose reductions required.

The patient subsequently underwent a follow-up prostate MRI, which revealed several notable findings. The prostate measured approximately 5.0×3.8×4.0 cm. The prostate volume was reduced compared to prior imaging studies. MRI demonstrated heterogeneous signal intensity with multiple nodular lesions of variable size in the central and transition zones. These lesions appeared isointense on T1WI and mildly hyperintense on T2WI. Notably, within the right transition zone, nodular abnormalities were identified between the 6 and 11 o’clock positions. The peripheral capsule appeared disrupted with ill-defined margins in this region. On imaging, this area showed isointense signal on T1WI, hypointense signal on T2WI, and mildly increased signal on DWI. Dynamic contrast-enhanced MRI revealed early and marked enhancement of the lesion, although the enhancement pattern was heterogeneous. The right peripheral zone lesion measured 3.5 × 2.1 cm, indicating a reduction in size from the previous measurement of 3.8 × 2.8 cm. No pelvic effusion was detected; however, small lymph nodes were observed in the pelvis and bilateral inguinal regions (**Figure 1E–H**).

**Figure 2:**
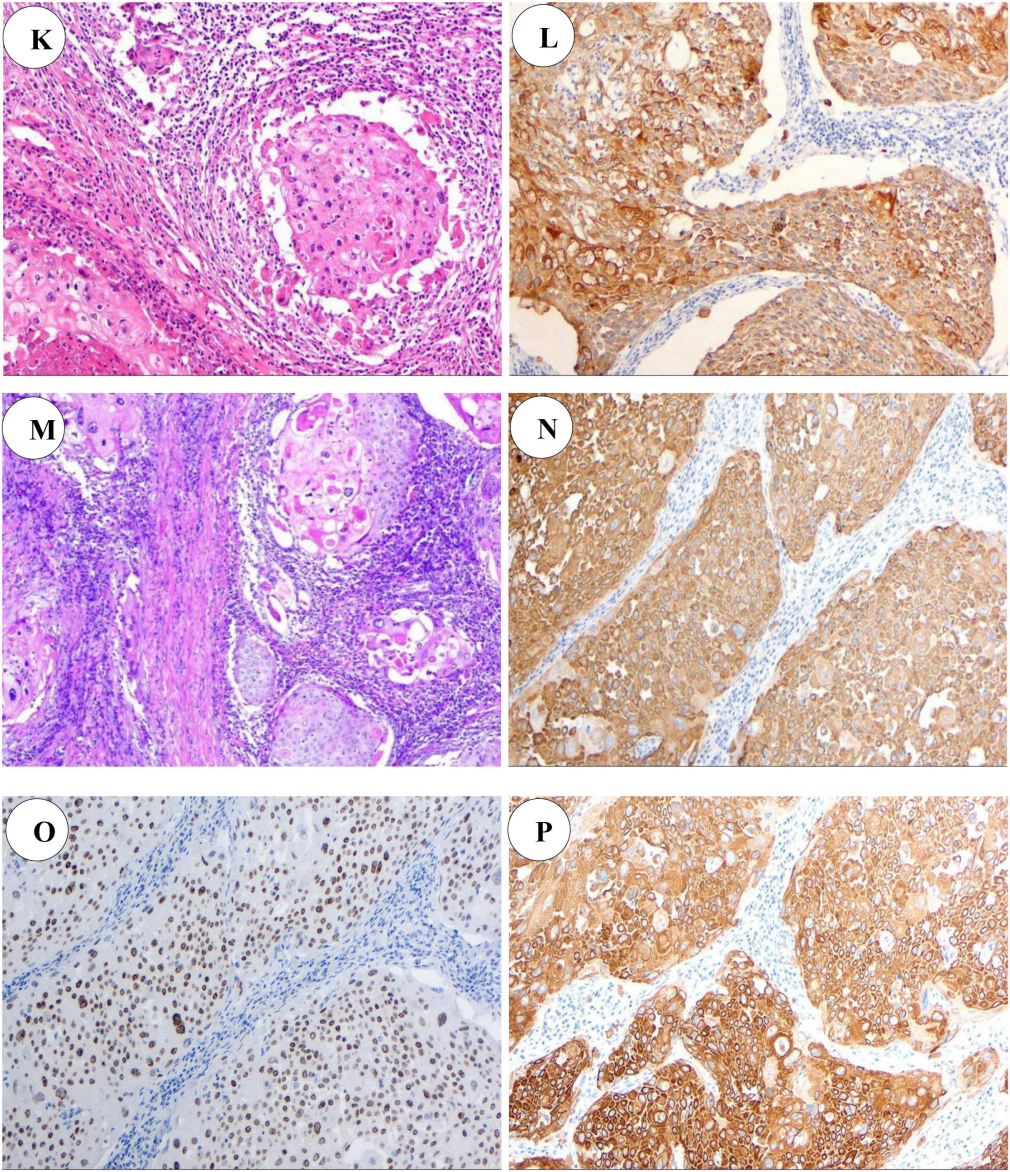
Pathologic manifestations of the patient **(K)** HE staining of left inguinal lymph node(100×); **(L)** Immunohistochemical staining of left inguinal lymph node CK5/6(+)(100×); **(M)** HE staining of radical prostatectomy specimen(100×); **(N)** Immunohistochemical staining of radical prostatectomy specimen CK5/6(+)(100×); **(O)** Immunohistochemical staining of prostate puncture specimen P40(+)(100×); **(P)** Immunohistochemical staining of prostate puncture specimen CKAE1/AE3(+)(100×).

Following chemotherapy, the patient’s MRI demonstrated a significant reduction in prostatic volume, decreasing from 59.7 mL to 39.6 mL—a reduction of approximately 33.7%. Given the patient’s strong preference for surgical intervention, a laparoscopic radical prostatectomy with extended pelvic lymph node dissection (ePLND) via an extraperitoneal approach was performed. Intraoperative assessment revealed dense fibro-adhesions involving the prostatic apex and base, necessitating meticulous dissection. The specimen was resected en bloc using an extracapsular technique, encompassing the prostate gland and bilateral seminal vesicles. Bilateral neurovascular bundle (NVB) preservation was not performed due to tumor proximity and intraoperative concern for margin compromise. Lymphadenectomy included all nodes within the standard ePLND template: external iliac, obturator, internal iliac, and presacral nodal groups bilaterally. Postoperative pathological examination revealed a moderately to poorly differentiated squamous cell carcinoma of the prostate, with focal necrosis, and interstitial lymphocytic infiltration. The tumor measured 1.6 × 1.5 × 0.8 cm and had invaded the bilateral seminal vesicles and periprostatic nerve bundles, with vascular invasion confirmed by the presence of tumor emboli. Notably, no tumor involvement was observed in the bilateral vas deferens, and the resection margins of the vas deferens were free of malignant cells. However, final pathology confirmed positive surgical margins at both the prostatic base and apex, with tumor cells extending to the inked resection surfaces and no residual benign prostatic parenchyma identified at these sites. Importantly, no metastatic deposits were detected in the bilateral pelvic lymph nodes. Immunohistochemical analysis showed diffuse positivity for CK AE1/AE3, CK5/6, p63, p40, and 34βE12, with focal expression of CK8/18. The markers P504S, PSA, GATA-3, AR, NKX3.1, S100P, Uroplakin II and C-erbB-2 were negative, while Ki-67 exhibited a proliferation index ranging from 20% to 75%, consistent with high proliferative activity (**Figure 2**). A follow-up PET-CT scan performed postoperatively revealed: No abnormal radiotracer accumulation was detected within the prostatectomy bed on postoperative imaging (**Figure 1J**).

Two months postoperatively, the patient received intensity-modulated radiotherapy (IMRT) targeting the prostate bed, elective pelvic lymph nodal regions, and bilateral inguinal basins. Image guidance was performed using daily cone-beam CT (CBCT). The prescribed dose to the planning gross tumor volume (PGTV)—encompassing macroscopic disease—was 55 Gy in 25 fractions (2.2 Gy per fraction), while the elective clinical target volume (CTV) was covered by a dose of 50 Gy in 25 fractions (2.0 Gy per fraction). No acute or subacute genitourinary or gastrointestinal toxicities ≥ grade 2 were observed during or within three months after treatment. Six months after surgery, a prostate MRI was performed, revealing irregular nodular lesions in the left pelvic region at the site of the previously resected seminal vesicles. These lesions measured approximately 2.1 × 1.6 cm and demonstrated isointense signal on T1WI, hyperintense signal on T2WI, and restricted diffusion with high signal intensity on DWI. Additionally, a patchy area of prolonged T2 signal was observed in the left pubic bone, exhibiting high signal intensity on DWI and enhancement on contrast-enhanced imaging, suggestive of possible osseous metastasis. At six months post–first-course RT, contrast-enhanced pelvic MRI revealed an irregular, enhancing nodular lesion in the left seminal vesicle fossa, consistent with local disease progression. In response, the patient underwent a second course of IMRT directed to the prostate bed and involved pelvic lymph nodes. Daily CBCT-based image guidance was again employed. For this re-irradiation, the PGTV (defined by the newly identified lesion and adjacent high-risk regions) received 66 Gy in 30 fractions (2.2 Gy per fraction), whereas the elective PTV (excluding previously irradiated low-risk nodal volumes) received 50 Gy in 25 fractions (2.0 Gy per fraction). Cumulative dose constraints for serial organs-at-risk—including bladder, rectum, bowel, and femoral heads—were rigorously evaluated using deformable image registration and adhered to institutional re-irradiation protocols. At 16 months postoperatively, follow-up MRI showed a reduction in the size of the nodular lesions in the left seminal vesicle bed, now measuring approximately 1.8 × 1.3 cm, with signal characteristics consistent with prior imaging. The prolonged T2 signal in the left pubis persisted, with continued DWI hyperintensity and contrast enhancement. Notably, the lesion in the left pelvic seminal vesicle region had markedly regressed, and the previously identified metastatic lymph nodes in the right inguinal region had completely resolved, indicating a favorable response to radiotherapy. The patient is currently undergoing treatment for bone metastases and remains alive, with nearly three years of continuous follow-up.

## 3 Discussion

### 3.1 Clinical features

Primary prostatic SCC is a rare clinical entity, accounting for approximately 0.5%–1% of all prostate cancer cases (3). As reported in the literature, patients with prostatic SCC typically present between the ages of 42 and 85 years and are frequently diagnosed at an advanced or metastatic stage, with a median overall survival of 12 to 14 months (5–8). prostatic SCC differs from conventional prostatic adenocarcinoma in multiple aspects, including cellular origin, biological behavior, response to standard hormonal therapy, and prognosis (7, 9). Biologically, prostatic SCC is more aggressive than adenocarcinoma, demonstrates limited responsiveness to conventional treatments, and is associated with a poorer clinical outcome. Clinically, however, prostatic SCC is indistinguishable from adenocarcinoma, commonly presenting with nonspecific lower urinary tract symptoms such as dysuria, urinary retention, hematuria, recurrent urinary tract infections, and bone pain secondary to metastasis. At initial diagnosis, approximately 56% of patients exhibit evidence of metastatic disease, which may be locoregional—invading the bladder neck, seminal vesicles, rectum, other pelvic organs, or pelvic lymph nodes—or distant, affecting sites such as bone, lung, and liver (5). Notably, bone metastases in prostatic SCC are predominantly osteolytic, contrasting with the osteoblastic lesions typically observed in prostatic adenocarcinoma. Therefore, histopathological confirmation is essential, and immunohistochemical analysis is routinely performed to rule out other pathological subtypes of prostate cancer (8).

The early diagnosis of prostatic SCC poses significant challenges compared to primary prostate adenocarcinoma, as the majority of patients present with metastatic disease at diagnosis. Lager et al. have demonstrated that prostatic SCC lacks the capacity to secrete PSA and prostatic acid phosphatase (PAP). As a result, serum levels of PSA and PAP typically remain within normal limits despite extensive tumor burden, thereby complicating differential diagnosis. This diagnostic ambiguity often leads to misdiagnosis, with some patients initially classified as having benign prostatic hyperplasia (5, 10, 11). Squamous cell carcinoma antigen (SCCA) is an established biomarker in the diagnosis and differentiation of malignancies arising from squamous epithelium, including cervical cancer, non-small cell lung cancer, head and neck cancer, esophageal cancer, and vulvar cancer. SCCA is predominantly localized in the cytoplasm of squamous cell carcinoma cells and is minimally expressed in normal tissues, but its expression is markedly upregulated in malignant epithelial lesions. Furthermore, SCCA plays a valuable role in monitoring treatment response, disease recurrence, metastasis, and prognosis. Notably, elevated serum SCCA levels have been observed in certain patients with prostatic SCC, suggesting its potential utility as a diagnostic and prognostic marker for this rare entity, as reported in the literature (12, 13). Given the rarity of prostatic SCC, further investigation through large-scale, multicenter studies with expanded sample sizes is warranted to validate these observations and establish clinical relevance.

### 3.2 Histopathological features

The precise histogenetic origin of prostatic SCC remains a subject of ongoing debate. Some researchers propose that prostatic SCC arises from the urothelium lining the prostatic urethra, whereas others suggest an origin from the urothelium of the periurethral ducts or from basal cells within the prostatic acini (10, 13). Notably, prostatic SCC may be histologically mistaken for benign squamous metaplasia, a non-neoplastic condition commonly observed adjacent to infarcted areas, in the setting of chronic inflammation or irritation, or following estrogen therapy. Moreover, other neoplastic conditions—such as urothelial carcinoma with squamous differentiation or adenocarcinoma exhibiting squamous metaplasia after hormonal treatment—may be misdiagnosed as primary prostatic SCC (3). Evidence suggests that squamous metaplasia in the prostate originates from basal cells that express nuclear estrogen receptors (14). Under conditions of excessive stimulation, particularly by estrogen, these receptor-positive basal cells undergo hyperproliferation and subsequent squamous differentiation. Thus, basal cells are considered potential progenitor cells in the development of prostatic SCC, especially in cases associated with estrogen exposure. These cells share immunohistochemical profiles with squamous cell carcinoma, including expression of markers such as high molecular weight cytokeratins and p63, further supporting their pathogenic role in prostatic SCC (15).

In 1979, Professor Mott established the first internationally recognized histopathological diagnostic criteria for prostatic SCC. These criteria include several key features: (I) the presence of characteristic malignant histological features, such as invasive growth, architectural disarray, and intercellular atypical differentiation; (II) definitive squamous morphological characteristics, including keratinization, formation of keratin pearls, and/or abundant intercellular bridges; (III) absence of glandular or acinar structures, which aids in differentiating prostatic SCC from adenocarcinoma with squamous metaplasia; (IV) no history of estrogen therapy; and (V) exclusion of a primary squamous cell carcinoma in the bladder or other extraprostatic sites. These criteria have played a critical role in the accurate diagnosis and distinction of prostatic SCC from histologically similar lesions (3).

### 3.3 Imaging features

Imaging modalities such as transrectal color Doppler ultrasound, CT, and MRI in patients with prostatic SCC often lack specific findings, making differentiation from prostatic adenocarcinoma challenging. As a result, a definitive diagnosis based on imaging alone remains difficult. However, MRI plays a pivotal role in detecting bone metastasis in prostate cancer. In prostatic SCC, bone metastases are predominantly osteolytic, appearing as hypointense signals on T1-weighted images and hyperintense signals on T2-weighted images. In contrast, prostatic adenocarcinoma typically presents with osteoblastic or mixed sclerotic lesions in bone metastases. Beyond conventional CT and MRI, PET-CT offers distinct potential and advantages in the detection and staging of malignant tumors. Although extensive research has been conducted on PET-CT performance in prostatic adenocarcinoma both domestically and internationally, detailed reports on its application in prostatic SCC remain limited (16). Previous studies in lung and esophageal cancers have demonstrated differential 18F-FDG uptake between adenocarcinoma and squamous cell carcinoma. Specifically, squamous cell carcinomas exhibit higher 18F-FDG accumulation compared to adenocarcinomas, which is attributed to increased expression of glucose transporter-1 (Glut-1) in squamous epithelial malignancies (17, 18).

Imperiale et al. demonstrated that 18F-FDG uptake is higher in esophageal squamous cell carcinomas than in adenocarcinomas. This uptake is influenced not only by histological type but also by the degree of tumor differentiation. Specifically, well-differentiated esophageal squamous cell carcinomas exhibit greater 18F-FDG uptake compared to poorly differentiated tumors. In contrast, poorly differentiated esophageal adenocarcinomas show reduced 18F-FDG uptake relative to their better-differentiated counterparts (19). The tissue origins and 18F-FDG avidities differ between prostatic SCC and prostate adenocarcinoma. Prostatic SCC is characterized by greater invasiveness, which is associated with increased cell proliferation, enhanced glucose metabolism, and upregulated expression of Glut-1 receptors. These factors contribute to increased 18F-FDG uptake in both the primary tumor and metastatic lesions, thereby facilitating differentiation from prostate adenocarcinoma to a certain extent (20, 21). Dong et al. reported that MRI T2-weighted images in patients with prostatic SCC revealed hyperintense lesions in the central region of the gland, whereas 18F-FDG PET-CT demonstrated marked tracer accumulation in tumor foci, with an SUVmax of 15.5. These findings may reflect the highly proliferative and invasive biological behavior of prostatic SCC (22). Kara et al. further suggested that prostatic SCC is associated with a more aggressive clinical course, characterized by increased proliferation of squamous carcinoma cells, which leads to enhanced glucose metabolism and upregulated expression of Glut-1. As a result, elevated 18F-FDG uptake is observed in both primary and metastatic lesions, with SUVmax measurements of 29.73 in the prostate and 11.40 in the right iliac perivascular lymph nodes, respectively. In contrast, primary prostate adenocarcinoma typically demonstrates relatively low 18F-FDG avidity in both the primary tumor and metastatic sites (20). Nevertheless, it should be noted that 18F-FDG PET-CT has limited sensitivity in detecting bone and lymph node metastases in prostate cancer (23).

Given that prostatic SCC exhibits distinct FDG uptake patterns compared to prostate adenocarcinoma, 18F-FDG PET-CT serves as an effective and non-invasive diagnostic modality for prostatic SCC. However, its widespread clinical application remains limited due to high costs.

### 3.4 Treatment protocols

The scarcity of reported cases of prostatic SCC in the literature has led to a lack of consensus on the optimal treatment regimen. Currently, both domestic and international experts generally agree that prostatic SCC demonstrates low sensitivity to endocrine therapy. As a result, early radical surgery, typically combined with chemotherapy and radiotherapy, is considered the most effective approach for managing primary prostatic SCC. In patients with distant organ metastases, a combination or alternating regimen of palliative surgery, chemotherapy, and radiotherapy is commonly adopted. This multimodal therapeutic strategy may modestly extend patient survival (3, 10, 13, 22, 24–28).

Surgical interventions, including radical prostatectomy, cystoprostatectomy, pelvic lymph node dissection, and metastasectomy, serve dual roles in the management of select prostate cancer cases. First, they may be utilized as primary treatment modalities for patients with localized or limited-stage disease. Second, these procedures can function as salvage therapies following recurrence of localized metastases. The literature reports several instances of long-term survival among patients who have undergone such surgical interventions, underscoring their potential therapeutic value in specific clinical contexts (28). A substantial body of evidence indicates that the combination of chemotherapy and radiotherapy is an effective strategy for prostatic SCC, significantly improving survival outcomes. Nevertheless, a standardized and prostatic SCC-specific chemotherapy regimen has not yet been established. Current chemotherapeutic protocols are primarily adapted from regimens used for squamous cell carcinomas in other anatomical sites, such as those in the head and neck region (8). Radiotherapy represents a viable option for localized tumors, and its use in prostatic SCC management shows promise-particularly when integrated with systemic therapy. This multimodal approach may enhance local tumor control and contribute to organ function preservation. However, the role of radiotherapy as an adjuvant treatment following surgery remains poorly defined and warrants further investigation. Therefore, additional research is required to fully elucidate its efficacy and optimal application in this setting (24).

Onoda et al. reported a case of locally advanced prostatic SCC with regional lymph node metastasis. The patient received seven cycles of the DCF chemotherapy regimen, consisting of 5-fluorouracil (5-FU) at 600 mg/m²/day on days 1–5, doxorubicin at 50 mg/m² on day 2, and cisplatin at 60 mg/m² on day 2, administered every four weeks. Concurrently, radiotherapy was delivered to the whole pelvis and prostate, with a total dose of 64 Gy. The treatment was well tolerated. Post-treatment imaging showed marked reduction in both primary tumor volume and enlarged lymph nodes. A subsequent prostate biopsy revealed no evidence of residual malignancy. During a continuous 24-month follow-up period, no disease recurrence was observed (12). Okada et al. described a case of prostatic SCC staged as T3N1M0 who underwent pelvic radiotherapy delivering a total dose of 50 Gy, with an additional 10 Gy boost to the prostate. Concurrent chemotherapy included two cycles of a regimen comprising peplomycin and cisplatin. Post-therapeutic evaluation demonstrated a reduction in prostate volume to 33% of baseline, and the previously enlarged iliac lymph nodes normalized in size. No signs of tumor progression were detected during an 18-month follow-up (13). Muñoz et al. reported the outcome of a patient with prostatic SCC treated with combined chemoradiotherapy. The chemotherapy regimen, modeled after protocols used for anal cancer, consisted of cisplatin (75 mg/m²) on day 1 and 5-FU (750 mg/m²) on days 1–5, repeated over three cycles. This was followed by definitive radiotherapy using a conventional four-field technique, delivering 46 Gy to the entire pelvis, an additional 20 Gy to the prostate bed, and a supplementary 6 Gy to the prostate, resulting in a cumulative dose of 72 Gy. The patient remained disease-free for five years before developing a localized recurrence (24). Evidence from the literature indicates that DCF regimens (doxorubicin, cisplatin, and 5-FU) are associated with improved survival in patients with head and neck cancer compared to standard CF regimens (cisplatin and 5-FU) (29). Onoda’s findings suggest that the integration of DCF chemotherapy with radiotherapy may represent a feasible therapeutic option for prostatic SCC, offering potential benefit for patients with advanced, inoperable disease (12).

## 4 Conclusion

In conclusion, prostatic SCC is a rare and highly aggressive variant of prostate cancer, posing significant diagnostic challenges due to nonspecific clinical manifestations and a high propensity for early metastasis. Early diagnosis and prompt initiation of treatment are critical for improving survival outcomes. However, differentiating prostatic SCC from conventional prostate adenocarcinoma and benign prostatic hyperplasia in the early stages remains difficult, as standard imaging modalities often lack sufficient specificity. Therefore, patients presenting with abnormal digital rectal examination findings but normal serum PSA levels should be closely monitored and considered for additional diagnostic evaluations. These may include assessment of alternative tumor markers, 18F-FDG PET-CT imaging, and prostate biopsy, to facilitate an accurate and timely diagnosis. Given the limited efficacy of endocrine therapy in prostatic SCC, early surgical intervention combined with concurrent chemotherapy and radiotherapy has been shown to significantly prolong survival and improve clinical outcomes. Patients with malignant tumors of the urinary system—particularly rare entities such as primary prostatic squamous cell carcinoma—derive significant clinical benefit from integrated, multidisciplinary management involving urology, anatomic pathology, and medical oncology (30). The rising global incidence of prostate cancer highlights the urgent need to enhance understanding of this rare subtype and refine its management strategies. Increased clinical awareness and intensified research efforts are essential to support the development of standardized treatment protocols for prostatic SCC. This case report aims to contribute to the existing body of knowledge and serve as a reference for future clinical and investigative work.

## Data Availability

The original contributions presented in the study are included in the article/supplementary material. Further inquiries can be directed to the corresponding author.
